# A fast and efficient count-based matrix factorization method for detecting cell types from single-cell RNAseq data

**DOI:** 10.1186/s12918-019-0699-6

**Published:** 2019-04-05

**Authors:** Shiquan Sun, Yabo Chen, Yang Liu, Xuequn Shang

**Affiliations:** 1 School of Computer Science, Northwestern Polytechnical University, Xi’an, Shaanxi, 710129 People’s Republic of China; 2 Key Laboratory of Big Data Storage and Management, Northwestern Polytechnical University, Ministry of Industry and Information Technology, Xi’an, Shaanxi, 710129 People’s Republic of China; 3 Centre for Multidisciplinary Convergence Computing (CMCC), School of Computer Science, Northwestern Polytechnical University, Xi’an, Shaanxi, 710129 People’s Republic of China; 4 Department of Biostatistics, University of Michigan, Ann Arbor, MI 48109 USA

**Keywords:** Single-cell RNA sequencing, Matrix factorization, Read count, Deep learning

## Abstract

**Background:**

Single-cell RNA sequencing (scRNAseq) data always involves various unwanted variables, which would be able to mask the true signal to identify cell-types. More efficient way of dealing with this issue is to extract low dimension information from high dimensional gene expression data to represent cell-type structure. In the past two years, several powerful matrix factorization tools were developed for scRNAseq data, such as NMF, ZIFA, pCMF and ZINB-WaVE. But the existing approaches either are unable to directly model the raw count of scRNAseq data or are really time-consuming when handling a large number of cells (e.g. *n*>500).

**Results:**

In this paper, we developed a fast and efficient count-based matrix factorization method (single-cell negative binomial matrix factorization, scNBMF) based on the TensorFlow framework to infer the low dimensional structure of cell types. To make our method scalable, we conducted a series of experiments on three public scRNAseq data sets, brain, embryonic stem, and pancreatic islet. The experimental results show that scNBMF is more powerful to detect cell types and 10 - 100 folds faster than the scRNAseq bespoke tools.

**Conclusions:**

In this paper, we proposed a fast and efficient count-based matrix factorization method, scNBMF, which is more powerful for detecting cell type purposes. A series of experiments were performed on three public scRNAseq data sets. The results show that scNBMF is a more powerful tool in large-scale scRNAseq data analysis. scNBMF was implemented in R and Python, and the source code are freely available at https://github.com/sqsun.

## Background

Single-cell RNA-sequencing (scRNAseq) analysis plays an important role in investigating tumour evolution, and is more powerful to characterize the intra-tumor cellular heterogeneity [1, 2]. Compared with traditional RNA sequencing (i.e. bulk RNAseq) which measures the specific gene expression level within a cell population, scRNAseq quantifies the specific gene expression level within only an individual cell [3, 4]. scRNAseq is more likely to understand the detailed biological processes of cell developmental trajectories and cell-to-cell heterogeneity, providing us fresh insights into cell composition, dynamic cell states, and regulatory mechanisms [5–8].

However, there are still several big challenges we have to carefully deal with before analyzing scRNAseq data [9, 10]. The first challenge is that the scRNAseq data is easy to involve some unwanted variables [11, 12], e.g. batch effects, confounding factors, etc. Moreover, the scRNAseq data set has their own characterizes, such as gene expression matrix is extremely sparse because of the quite small number of mRNAs represented in each cell [13]; current sequencing technologies, e.g. CEL-Seq2 [14] and Drop-seq [15], etc, do not have enough power to quantify the actual concentration of mRNAs (i.e. well-known “dropout events”) [16]; the heavy amplifications may result into strong amplification bias [17]; cell cycle state, cell size or other unknown factors may contribute to cell-cell heterogeneity even within the same cell type [18].

The second important feature of the scRNAseq data set is of count nature [19]. In most RNA sequencing studies, the number of reads mapped to a given gene or isoform is often used as an intuitive estimate of its expression level. To account for the count nature of the RNA sequencing data, and the resulting mean-variance dependence, most statistical methods were developed using discrete distributions in differential expression analysis, i.e., PQLseq [20], edgeR/DESeq [21, 22], and MACAU [23]. Therefore, a nature choice of analyzing scRNAseq data is to develop count-based dimensionality reduction methods. Although several dimensionality reduction techniques have been already applied to scRNAseq data analysis, such as principal component analysis (PCA) [24]; independent components analysis (ICA) [25], and diffusion map [26]; partial least squares (PLS) [27, 28]; nonnegative matrix factorization (or factor analysis) [29, 30], gene expression levels are inherently quantified by counts, i.e., count nature of scRNAseq data [31, 32].

Therefore, developing the bespoke scRNAseq dimensionality reduction method has been triggered within the last two years. The first factor analysis method, ZIFA, is trying to model the drop-out events via the zero-inflated model, but the method does not take into account the count nature of the data [33]; pCMF is trying to build sparse Gamma-Poisson factor model within the Bayesian framework, but such method does not include the covariates [34]; ZINB-WaVE is trying to involve both gene-level and sample-level covariates via a hierarchical model, but the method is really time-consuming when sample size is large [35, 36].

**Fig. 1:**
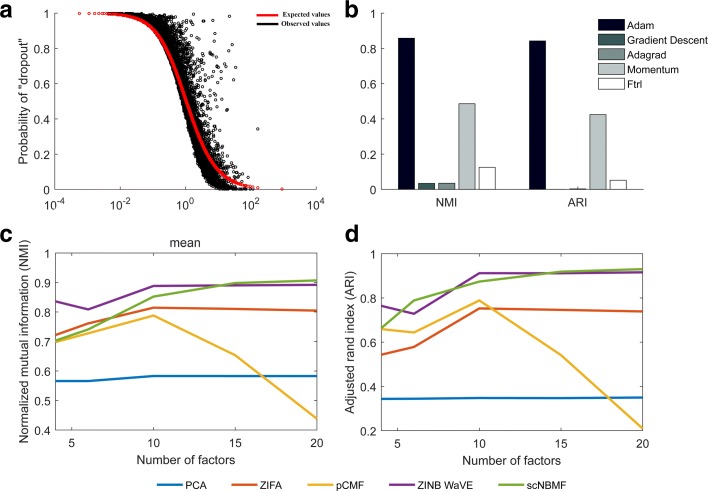
A simple example to show the parameter effect or optimizer effect of NMI and ARI in scRNA-seq data on clustering. **a** This figure shows the relationship between mean gene expression levels and dropout rates. The black line indicates observed value, which is computed by the number of unexpressed cells divided by the number of cells; The red line represents expected value, which is calculated by negative binomial distribution with mean gene expression levels and dispersion parameter *ψ*(*ψ*=*mean*(*ψ*_*i*_))**b** This figure shows how optimizers affect the performance of different methods on NMI and ARI. **c**-**d** These two figure indicate how the number of factors affect the NMI and ARI, respectively

Here, in this paper, we propose a fast and efficient count-based matrix factorization method that utilizes the negative binomial distribution to account for the over-dispersion problem of the count nature of scRNAseq data, single-cell Negative Binomial-based Matrix Factorization, scNBMF. The reason of choosing negative binomial model instead of zero-inflated negative binomial model is that not only the most scRNAseq data sets do not show much technical contribution to zero-inflation (Fig. 1a), but also can largely reduce the computation burden in estimating drop-out parameters for each gene. With the stochastic optimization method Adam [37] implemented within TensorFlow framework, scNBMF is roughly 10 – 100 times faster than the existing count-based matrix factorization methods, such as pCMF and ZINB-WaVE. To make the proposed method scalable, we apply scNBMF to analyze three publicly available scRNAseq datasets. The results demonstrate that scNBMF is more efficient and powerful than other matrix factorization methods.

## Materials and methods

### scNBMF: model and algorithm

scNBMF is to fit the logarithm likelihood function of negative binomial model-based matrix factorization. Given *n* cells and *p* genes, we denote *Y* as a gene expression matrix, and its element *y*_*ij*_ is the count of gene *i* and cell *j*. To account for the over-dispersion problem, we model the gene expression level *y*_*ij*_ as a random variable following the negative binomial distribution with parameters *μ*_*ij*_ and *ϕ*_*i*_, i.e.,

$$y_{ij} \sim NB(\mu_{ij},\phi_{i}) $$

where the rate parameter *μ*_*ij*_ denotes the mean expression level for gene *i* and cell *j*; the parameter *ϕ*_*i*_ represents variance of gene expression, typically means gene-specific over-dispersion; *NB* is the negative binomial distribution, i.e.

$$ \text{Pr}_{NB}(y_{ij}|\mu_{ij},\phi_{i}) \,=\, \left(\! \begin{array}{c} y_{ij} + \phi_{i} - 1\\ y_{ij} \end{array} \!\!\right) \!\!\left(\frac{\mu_{ij}} {\mu_{ij} + \phi_{i} } \!\right)^{y_{ij}}\!\! \left(\frac{ \phi_{i}} {\mu_{ij} + \phi_{i}} \!\right)^{\phi_{i}}\!. $$

For the rate parameter *μ*_*ij*_, we consider the following regression model

$$log(\mu_{ij}) = log(N_{j}) + {\sum}_{k = 1}^{K} W_{ik} X_{kj}. $$

where *N*_*j*_ is the total read count for the individual cell *j* (a.k.a read depth or coverage); *W*_*ik*_ is the loadings while *H*_*kj*_ is the factors represents the coordinates of the cells, which can be used to identify cell type purpose; *K* is the pre-defined number of components; When all *ϕ*_*i*_→0, the negative binomial distribution will reduce to the standard Poisson distribution.

Therefore, the log-likelihood function for gene *i* and cell *j* is

$$\begin{array}{*{20}l} \mathcal{L}_{NB}(\mu,\phi| Y) = &\sum\limits_{i = 1}^{p} \sum\limits_{j = 1}^{n} log \text{Pr}_{NB} \left(y_{ij}|\mu_{ij},\phi_{i}\right)\\ = &\sum\limits_{i = 1}^{p} \sum\limits_{j = 1}^{n} y_{ij} log (\mu_{ij}) + \phi_{i} log(\phi_{i}) \\&- (y_{ij} + \phi_{i}) log (\mu_{ij}+\phi_{i}) \\ & + log\left(\begin{array}{c} y_{ij} + {\phi_{i}} - 1\\ y_{ij} \end{array} \right). \end{array} $$

where *μ* denotes the mean gene expression matrix and its element $\mu _{ij}=e^{log(N_{j}) + {\sum }_{k = 1}^{K} W_{ik} X_{kj} }$; *ϕ* is a *p*-vector, and its element *ϕ*_*i*_ represents the over-dispersion parameter for gene *i*.

To make our model more interpretation for the biological applications, we introduce a sparse penalty (LASSO) on loading matrix *W* since some genes are expressed while some are not in real-world biological processes. Therefore, the objective function of optimization problem becomes

$$\mathcal{L} = \mathcal{L}_{NB}(\mu,\phi|Y) + \lambda \sum\limits_{i = 1}^{p} \left\| W_{i} \right\|_{1} $$

where ∥·∥_1_ is a *l*_1_-norm (i.e. LASSO penalty); *λ* denotes the penalty parameter.

In the above model, we are interested in extracting the factor matrix *H* for detecting the cell type purposes. We first estimate the dispersion parameter *ϕ*_*i*_) for each gene via edgeR [21] with default parameter settings, then fit the above model using Adam optimizer within TensorFlow. For deep learning model, we set the learning rate of the network as 0.001 and maximum iteration as 18000.

### Compared methods and evaluations

To make scNBMF scalable, we compared seven existing methods, i.e. PCA, Nimfa, NMFEM, tSNE, ZIFA, pCMF, and ZINB-WaVE, in the experiments. Since PCA and ZIFA are only for normalized gene expression data, we normalized raw count data following previous recommendations [38]. Typically, we transformed the count data using base 2 and pseudo count 1.0, i.e., *log*_2_(*Y*+1.0), into continuous data. The performance of each method was evaluated by the normalized mutual information (NMI), defined in [39]

1 $$ NMI(L_{e}, L) = \frac{\sum\limits_{k = 1}^{K}\sum\limits_{t = 1}^{K_{e}} \frac{n_{kt}}{n}log \left(\frac{n_{kt}}{n} \right) - \sum\limits_{k = 1}^{K}\frac{n_{k}}{n}log \left(\frac{n_{k}}{n} \right)- \sum\limits_{t = 1}^{K_{e}}\frac{n_{t}}{n}log \left(\frac{n_{t}}{n} \right)} {\sqrt{\sum\limits_{k = 1}^{K}\frac{n_{k}}{n}log \left(\frac{n_{k}}{n} \right)* \sum\limits_{t = 1}^{K_{e}}\frac{n_{t}}{n}log \left(\frac{n_{t}}{n}\right)} }.  $$

and the adjusted rand index (ARI), defined in [40]

$$ ARI(L_{e}, L) = \frac{{{\sum}_{kt} {\left(\begin{array}{l} n_{kt}\\ 2 \end{array} \right) - \left({{\sum}_{k} {\left(\begin{array}{l} n_{k}\\ 2 \end{array} \right) {\sum}_{t} {\left(\begin{array}{l} n_{t}\\ 2 \end{array} \right)}} } \right)/\left(\begin{array}{l} n\\ 2 \end{array} \right)}}}{{\frac{1}{2}\left({{\sum}_{k} {\left(\begin{array}{l} n_{k}\\ 2 \end{array} \right) + {\sum}_{t} {\left(\begin{array}{l} n_{t}\\ 2 \end{array} \right)}} } \right) - \left({{\sum}_{k} {\left(\begin{array}{l} n_{k}\\ 2 \end{array} \right){\sum}_{t} {\left(\begin{array}{l} n_{t}\\ 2 \end{array} \right)}} } \right)/\left(\begin{array}{l} n\\ 2 \end{array} \right)}}. $$

where *L*_*e*_ and *L* are the predicted cluster labels and the true labels, respectively; *K*_*e*_ and *K* are the predicted cluster number and the true cluster number, respectively; *n*_*k*_ denotes the number of cells assigned to a specific cluster *k* (*k*=1,2,⋯,*K*); similarly *n*_*t*_ denotes the number of cells assigned to cluster *t* (*t*=1,2,⋯,*K*_*e*_); *n*_*kt*_ represents the number of cells shared between cluster *k* and *t*; and *n* is the total number of cells.

### Public scRNAseq data sets

Three publicly available scRNAseq data sets were collected from three studies:

- The first scRNAseq data set was collected from human brain [41]. There are 420 cells in eight cell types after excluded hybrid cells including, fetal quiescent cells (110 cells), fetal replicating cells (25 cells), astrocytes cells (62 cells), neuron cells (131 cells), endothelial (20 cells) and oligodendrocyte cells (38 cells) microglia cells(16 cells), and (OPCs, 16 cells), and remain 16,619 genes to test after filtering out the lowly expressed genes. The original data was downloaded from the data repository Gene Expression Omnibus (GEO; GSE67835);
- The second scRNAseq data set was collected from human pancreatic islet [42]. There are 60 cells in six cell types after excluding undefined cells including alpha cells (18 cells), delta cells (2 cells), pp cells (9 cells), duct cells (8 cells), beta cells (12 cells) and acinar cells (11 cells),and 116,414 genes to test after filtering out the lowly expressed genes. The original data was downloaded from the data repository Gene Expression Omnibus (GEO; GSE73727);
- The third scRNAseq data set was collected from the human embryonic stem [43]. There are 1018 cells which belong to seven known cell subpopulations that include neuronal progenitor cells (NPCs, 173 cells), definitive endoderm derivative cells (DEDs), endothelial cells (ECs, 105 cells), trophoblast-like cells (TBs, 69 cells), undifferentiated H1(212 cells) and H9(162 cells) ESCs, and fore-skin fibroblasts (HFFs, 159 cells), and contains 17,027 genes to test after filtering step. The original data was downloaded from the data repository Gene Expression Omnibus (GEO; GSE75748).

## Results

### Model selection

Our first set of experiments is to select the optimization method for the log-likelihood function of negative binomial matrix factorization model. Without loss of generality, we choose the human brain scRNAseq data set. Five optimization methods were compared to optimize the neural networks, i.e., Adam, gradient descent, Adagrad, Momentum and Ftrl. The results show that the Adam significantly outperforms other optimization methods regardless of what criteria we choose (Fig. 1b). Specifically, for NMI, Adam, gradient descent, Adagrad, Momentum, and Ftrl achieve 0.8579, 0.0341, 0.0348, 0.4859, and 0.1251, respectively. Therefore, in the following experiments, we will choose the Adam method to optimize the neural networks.

Our second set of experiments is to select the number of factors in the low dimensional structure of cell types. Without loss of generality, we still choose the human brain scRNAseq data set. We varied the number of factors (*k* = 4, 6, 10, 15, and 20). The results demonstrate that the number of factors does not impact PCA (Fig. 1c and d; bule line). The other four methods show an increasing pattern when the number of factors varied from 4 to 20 (Fig. 1c and d). Therefore, we choose the top 20 factors in the following experiments.

### Public scRNAseq data sets

Our third set of experiments is to apply scNBMF to three scRNAseq real data sets, human brain, human pancreas islet, and human embryonic stem. The cell type information of the three data sets were reported by the original studies. For the comparison, we compared seven other methods, PCA, Nimfa, NMFEM, tSNE, ZIFA, pCMF and ZINB-WaVE. For the evaluation, we extracted the low dimensional structure with top 10 factors, and used *k*-means clustering method in an unsupervised manner, repeated 100 times to test how well each method can recover the cell type assignments on NMI and ARI in the studies.

**Fig. 2:**
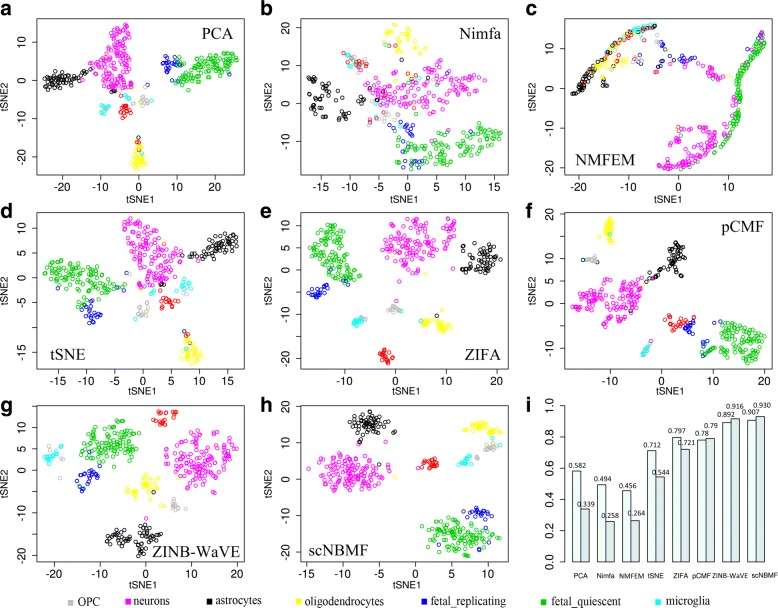
Performance evaluation on human brain scRNA-seq data. In this data set there are 420 cells in eight different cell types after the exclusion of hybrid cells. Each kind of color represent a kind of cell type. **a**-**h** These eight figures display the clustering output of two dimension of tSNE using eight matrix factorization methods(PCA, Nimfa, NMFEM, tSNE, ZIFA, pCMF, ZINB-WaVE, and scNBMF). **f** This figure shows NMI and ARI values which are from eight compared methods

The first biological data application is performed on the human brain scRNAseq data set. Figure 2 demonstrates the comparison results of tSNE with respect to seven compared clustering methods. scNBMF shows the clearly cell type patterns with the annotated cell type (Fig. 1h). Also, we carried out the same analysis using PCA (Fig. 2a), Nimfa (Fig. 2b), NMFEM (Fig. 2c), tSNE (Fig. 2d), ZIFA (Fig. 2e), pCMF (Fig. 2f), and ZINB-WaVE (Fig. 2g). For NMI and ARI, scNBMF outperforms the other methods. Specifically, for NMI criterion, PCA, Nimfa, NMFEM, tSNE, ZIFA, pCMF, ZINB-WaVE and scNBMF achieve, 0.582, 0.494, 0.456, 0.712, 0.797, 0.787, 0.892, and 0.901, respectively (Fig. 2i and Table 1); while for ARI criterion, PCA, Nimfa, NMFEM, tSNE, ZIFA, pCMF, ZINB-WaVE and scNBMF achieve, 0.339, 0.258, 0.264, 0.544, 0.721, 0.788, 0.916, and 0.933, respectively (Fig. 2i and Table 1).

**Table 1:** Clustering comparison of the matrix factorization-based methods in terms of Normalized Mutual information (NMI) and Adjusted Random Index (ARI)

Method	Brain		Embryo		Pancreas	
	NMI	ARI	NMI	ARI	NMI	ARI
PCA	0.582	0.339	0.366	0.187	0.630	0.368
Nimfa	0.494	0.258	0.414	0.173	0.456	0.114
NMFEM	0.456	0.264	0.741	0.614	0.435	0.175
tSNE	0.712	0.544	0.658	0.538	**0.793**	**0.652**
ZIFA	0.797	0.721	0.888	0.748	0.641	0.429
pCMF	0.787	0.788	0.822	0.659	0.547	0.334
ZINB-WaVE	0.892	0.916	0.888	0.721	0.518	0.342
scNBMF	**0.901**	**0.933**	**0.908**	**0.763**	0.716	0.472

The number with bold indicates the best performance method and the number with grey represents the second best performance method

The second biological data application is to investigate the character of human pancreas islet scRNAseq data set. This data set has a smaller number of cells - only 60 cells in six cell types. Since all methods do not have enough power to detect the cell type clustering patterns, we did not show the tSNE plots for this data set. For NMI and ARI, tSNE shows the highest performance, while scNBMF achieves the second best performance (Table 1). Specifically, tSNE achieves 0.973 and 0.652 on NMI and ARI, respectively; while scNBMF is 0.716 and 0.472 on NMI and ARI respectively.

**Fig. 3:**
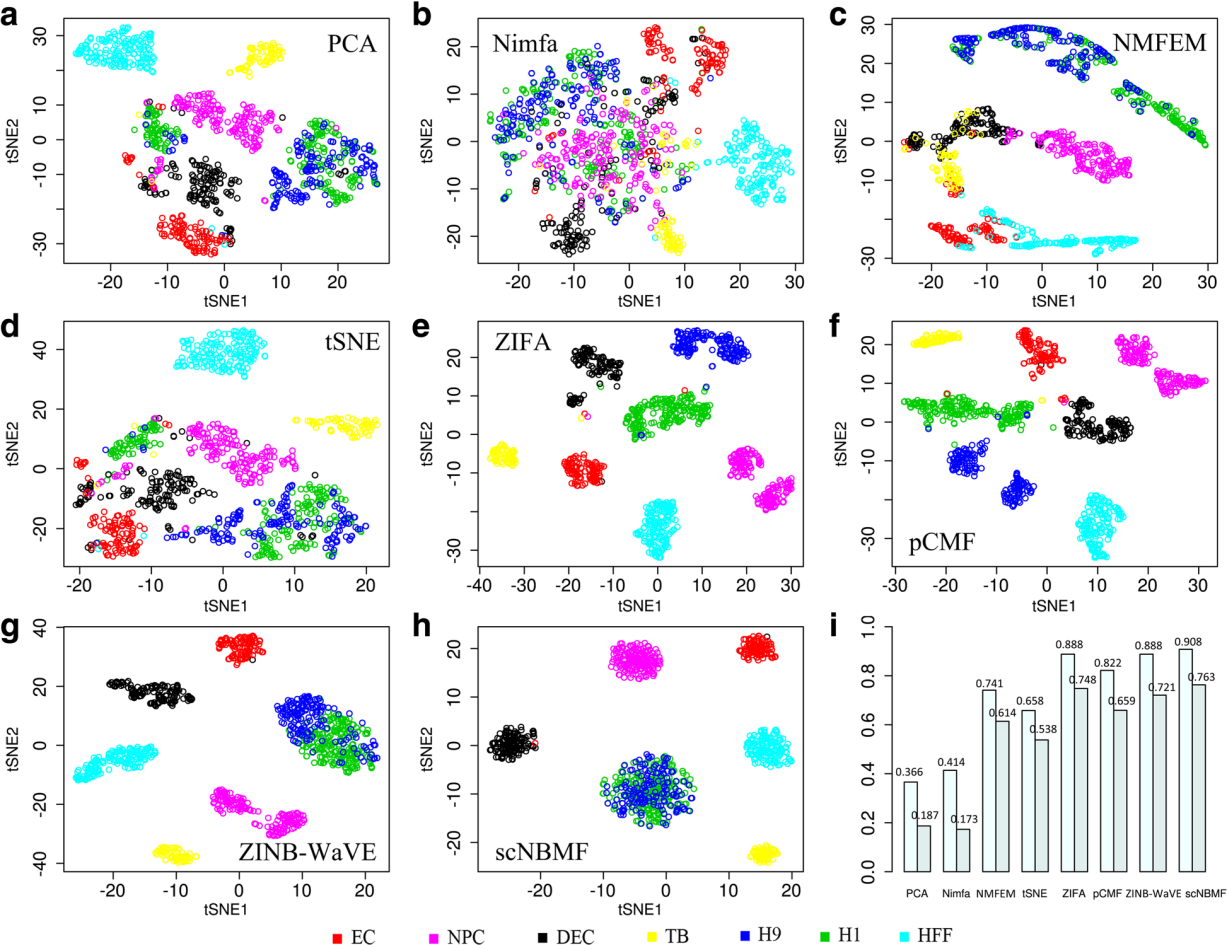
Performance evaluation on human embryonic stem scRNA-seq data set, which contains 1018 cells in seven cell types. Different colors also represent different cell types. **a**-**h** These five figure display the clustering output of two dimension of tSNE using five matrix factorization methods(PCA, Nimfa, NMFEM, tSNE, ZIFA, pCMF, ZINB-WaVE, and scNBMF). **f** This figure shows NMI and ARI values which are from eight compared methods

The third biological data application is to investigate lineage-specific transcriptomic features at single-cell resolution. To elucidate the distinctions between different lineages, we performed eight matrix factorization methods, i.e., PCA (Fig. 3a), Nimfa (Fig. 3b), NMFEM (Fig. 3c), tSNE (Fig. 3d), ZIFA (Fig. 3e), pCMF (Fig. 3f), ZINB-WaVE (Fig. 3g), and scNBMF (Fig. 3h). scNBMF demonstrates more clearly their respective cell-type patterns compared with other methods. The cell type H1 and H9 show the tight overlapping pattern to indicate the relative homogeneity of human ES cells, such results are also consistence with the previous results [43]. For NMI and ARI, scNBMF outperforms other methods (Fig. 3i and Table 1). Specifically, for NMI, PCA, Nimfa, NMFEM, tSNE, ZIFA, pCMF, ZINB-WaVE and scNBMF achieve, 0.366, 0.414, 0.741, 0.658, 0.888, 0.822, 0.888, and 0.908, respectively; For ARI, PCA, Nimfa, NMFEM, tSNE, ZIFA, pCMF, ZINB-WaVE and scNBMF achieve, 0.187, 0.173, 0.614, 0.538, 0.748, 0.659, 0.721, and 0.763, respectively.

### Computation time

The last set of experiments is to compare the computation time of PCA, Nimfa, NMFEM, tSNE, ZIFA, pCMF, and ZINB-WaVE. Without loss of generality, we use human brain data set to show the computation time of the compared methods (Table 2). Nimfa, NMFEM, ZIFA, pCMF, and ZINB-WaVE are the bespoke scRNAseq methods. Compared with the count-based methods, ZINB-WaVE and pCMF, scNBMF is roughly 100 folds faster than ZINB-WaVE, and 10 folds faster than pCMF. Even comparing the non-count based methods, ZIFA, Nimfa, and NMFEM, scNBMF is still the fastest method.

**Table 2:** Computation times (second) of the matrix factorization-based methods on human brain scRNAseq data set, *k* represents the number of factors

Method	*k*=2	*k*=4	*k*=6	*k*=10	*k*=15	*k*=20
PCA	11.54	11.55	11.70	11.35	11.37	11.59
Nimfa	639.15	1990.66	2260.13	2490.05	2705.42	2924.87
NMFEM	1471.39	1628.2	1913.11	2248.18	2659.23	3027.5
tSNE	1.85	14.41	32.11	56.01	77.20	101.25
ZIFA	5331.25	5831.04	6347.08	6987.52	7338.26	7722.33
pCMF	12391.6	13517.12	14260.26	15111.55	15978.44	17158.42
ZINB-WaVE	71053.1	79402.17	90118.3	101072.9	115379.7	126575.2
scNBMF	456.12	478.90	541.31	717.88	1053.22	1563.75

## Conclusion

With rapid developing sequencing technology, a large amount of scRNAseq data sets is easily obtained via different sources. Therefore, computation time is one of these big issues for downstream analysis. On the other hand, scRNAseq data have their own characterizes, i.e., count nature, noisy, and sparsity, etc. These have been triggered the development of a fast and efficient count-based matrix factorization method. In this paper, we proposed a count-based matrix factorization (scNBMF) method to model the raw count data, prevent losing information from normalizing raw count data. On three public biological scRNAseq data sets, scNBMF provides powerful performance compared with other seven methods in terms of NMI, ARI, and computation time.

Zero-inflated distribution is more appropriate method to account for dropouts, e.g. ZIFA and ZINB-WaVE. In current study, we did not consider the zero-inflated model because the tested data sets do not show too much dropouts. However, this is a necessary step in analyzing some scRNAseq data sets. Therefore, we will add the zero-inflated distribution in the future version of the scNBMF.

Biologically, if we incorporate all genes in scRNAseq data analysis, probably it would be able to involve some unwanted variables because not all genes are expressed in biological processes. An interesting direction to improve the performance of scNBMF is to select some informative genes first, this step can largely reduce unwanted variables, and exclude some redundancy genes [44, 45] in the downstream analysis. In addition, because gene expression levels are highly affected by other gene specific annotations, such as GC-content, gene length, and chromatin states [46]. If some interesting variables in the statistical model, such as “drop-out” parameter, can be inferred by annotation information, the method probably will significantly improve the power of detecting cell types from scRNAseq data.

## Abbreviations

ARI: Adjusted rand index
DESeq: Differential expression
edgeR: Empirical analysis of digital gene expression data in R
ICA: Independent components analysis
MACAU: mixed model association for count data via data augmentation
NMI: Normalized mutual information
PCA: Principal component analysis
pCMF: Probabilistic count matrix factorization
PLS: Partial least squares
PQLseq: Penalized quasi-likelihood
scNBMF: Single-cell negative binomial matrix factorization
scRNAseq: Single-cell RNA sequencing
tSNE: t-distributed stochastic neighbor embedding
ZIFA: Zero-inflated factor analysis
ZINB-WaVE: Zero-inflated negative binomial-based wanted variation extraction
